# Differential circular RNA expression profiles in umbilical cord blood exosomes from preeclampsia patients

**DOI:** 10.1186/s12884-021-03777-7

**Published:** 2021-04-15

**Authors:** Minkai Cao, Juan Wen, Chaozhi Bu, Chunyan Li, Yu Lin, Hong Zhang, Yanfang Gu, Zhonghua Shi, Yan Zhang, Wei Long, Le Zhang

**Affiliations:** 1 Department of Obstetrics, The Affiliated Wuxi Maternity and Child Health Care Hospital of Nanjing Medical University, Wuxi, 214002 China; 2 Nanjing Maternity and Child Health Care Institute, Women’s Hospital of Nanjing Medical University, Nanjing Maternity and Child Health Care Hospital, Nanjing, 210004 China; 3 Research Institute for Reproductive Medicine and Genetic Diseases, The Affiliated Wuxi Maternity and Child Health Care Hospital of Nanjing Medical University, Wuxi, 214002 China; 4 Department of Obstetrics, Women’s Hospital of Nanjing Medical University, Nanjing Maternity and Child Health Care Hospital, Nanjing, 210004 China; 5 Department of Neonatology, The Affiliated Wuxi Children’s Hospital of Nanjing Medical University, Wuxi, 214023 China

**Keywords:** Umbilical cord blood, Exosomes, Circular RNA, Preeclampsia

## Abstract

**Background:**

Exosomal circular RNAs (circRNAs) are emerging as important regulators of physiological development and disease pathogenesis. However, the roles of exosomal circRNAs from umbilical cord blood in preeclampsia (PE) occurrence remains poorly understood.

**Methods:**

We used microarray technology to establish the differential circRNA expression profiles in umbilical cord blood exosomes from PE patients compared with normal controls. Bioinformatics analysis was conducted to further predict the potential effects of the differentially expressed circRNAs and their interactions with miRNAs.

**Results:**

According to the microarray data, we identified 143 significantly up-regulated circRNAs and 161 significantly down-regulated circRNAs in umbilical cord blood exosomes of PE patients compared with controls. Gene Ontology (GO) and Kyoto Encyclopedia of Genes and Genomes (KEGG) biological pathway analyses showed that circRNA parental genes involved in the regulation of metabolic process, trophoblast growth and invasion were significantly enriched, which play important roles in PE development. Moreover, pathway network was constructed to reveal the key pathways in PE, such as PI3K-Akt signaling pathway. Further circRNA/miRNA interactions analysis demonstrated that most exosomal circRNAs had miRNA binding sites, and some miRNAs were associated with PE.

**Conclusions:**

Our results highlight the importance of exosomal circRNAs in the pathogenesis of PE and lay a foundation for extensive studies on the role of exosomal circRNAs in PE development.

**Supplementary Information:**

The online version contains supplementary material available at 10.1186/s12884-021-03777-7.

## Background

Preeclampsia (PE) is a gestation-specific syndrome that affects up to 5–7% of pregnancies, characterized by elevated blood pressure and proteinuria after 20 weeks of pregnancy [1, 2]. This multisystem pregnancy disorder is often accompanied by headache, nausea, vomiting, upper abdominal discomfort and other symptoms, and is a leading cause of maternal and neonatal morbidity and mortality worldwide [3]. Due to the multifactorial nature of the disorder, the exact etiology of PE remains largely unknown, and there is currently no effective treatment for PE other than termination of pregnancy. It is reported that PE and its related diseases are responsible for nearly 40% of premature births delivered before 35 weeks of gestation [4]. After giving birth, although most women return to normal blood pressure levels, PE reflects an elevated long-term risk of cardiovascular diseases in both the mother and the child [5–7].

Exosomes have become key mediators of local and systemic intercellular communication by regulating different biological processes between cells [8, 9]. Many studies have reported that exosomes play an important role in the regulation of pregnancy complications such as preeclampsia and gestational diabetes [10, 11]. Specific exosomes derived from placenta can be detected in maternal blood as early as the 6th week of gestation, and concentrations of placenta-derived exosomes increase as pregnancy progresses [12]. In addition, exosomes isolated from maternal blood are biologically active in vitro and can enter target cells through endocytosis [13]. Circular RNA (circRNA) is a novel member of endogenous noncoding RNAs, which is widely distributed and has a variety of cellular functions. Recently, circRNAs have been identified for their enrichment and stability in exosomes [14, 15]. More and more studies have shown that exosomal circRNAs are involved in the processes of cell growth, angiogenesis, epithelial mesenchymal transition and targeted therapy [16, 17]. And accumulating evidences suggested that it is possible to identify functional and/or structural differences in umbilical cord blood with risk of PE [18–20]. Therefore, we speculated that exosomal circRNA in umbilical cord blood might play an important role in the regulation of PE development as a new placental derived factor. However, the role of exosomal circRNAs from umbilical cord blood in PE development remains unclear. In this study, we used microarray technology to construct a comparative exosomal circRNA profiling of umbilical cord blood between PE patients and controls, aiming to lay a foundation for further research on the role of exosomal circRNAs in PE development.

## Methods

### Patients and sample collection

All participants and clinical information were collected at the Nanjing Maternity and Child Health Care Hospital and Wuxi Maternity and Child Health Care Hospital from September 2019 to February 2020. PE was defined as new-onset hypertension (blood pressure ≥ 140/90 mmHg on two separate occasions at least 6 h apart or blood pressure ≥ 160/110 mmHg) and proteinuria (> 300 mg/24 h) after 20 weeks of gestation in previously normotensive women. The pregnant women without PE were included as controls. The controls were matched to PE cases for maternal age. In order to reduce selection bias with different PE severity, pregnant women with gestational age greater than 34 weeks were included in this study. At last, 46 umbilical cord blood samples were collected from the umbilical vein immediately after delivery of fetus during cesarean section (23 PE patients and 23 controls) according to the standard operating procedure. All participants were divided into two sets, 6 participants (3 PE patients and 3 controls) as pilot sample for microarray screening and 40 participants (20 PE patients and 20 controls) for validation. The basic information of the 46 subjects was shown in Table S1.

### Purification and analysis of exosomes

Exosomes were extracted from the umbilical cord blood. In brief, umbilical cord blood was centrifuged at 3000 g for 15 min at 4 °C. Then supernatants were centrifuged at 12,000 g for 30 min at 4 °C. The supernatants were then filtered by a 0.45 μm polyvinylidene fluoride membrane, and finally isolated by ultracentrifugation at 100,000 g for 180 min at 4 °C. The exosome particles were resuspended in lysis buffer or PBS. The resulting exosomes were then analyzed using Nanosight Nano ZS device (Malvern Instruments, UK).

### Exosomal RNA extraction and microarray analysis

Total exosomal RNA was extracted using exoRNeasy Serum/Plasma Maxi Kit (QIAGEN, Germany) according to the manufacturer’s instructions and checked for RNA integrity by an Agilent Bioanalyzer 2100 (Agilent technologies, USA). Total RNA was amplified and labeled by Low Input Quick Amp Labeling Kit (Agilent technologies, USA), following the manufacturer’s instructions. Labeled cRNA was purified by RNeasy mini kit (QIAGEN, Germany). Each slide was hybridized with 1.65 μg Cy3-labeled cRNA using Gene Expression Hybridization Kit (Agilent technologies, USA) in Hybridization Oven (Agilent technologies, USA), according to the manufacturer’s instructions. After 17 h hybridization, slides were washed in staining dishes (Thermo Shandon, USA) with Gene Expression Wash Buffer Kit (Agilent technologies, USA), followed the manufacturer’s instructions. Slides were scanned by Agilent Microarray Scanner (Agilent technologies, USA) with default settings. Data were extracted with Feature Extraction software 12.0 (Agilent technologies, USA). Raw data were normalized by Quantile algorithm, limma packages in R.

### Quantitative real-time PCR (qPCR)

After the total exosomal RNA was extracted and RNA integrity was checked according to the above method, RNA quantity and quality were measured with a Nano Drop ND-1000 spectrophotometer (Thermo, USA). Then, the RNA was digested using RNase R (Epicenter Biotechnologies, USA) and purified. Complementary DNA was acquired from reverse transcription of 500 ng RNA using PrimeScript RT reagent with gDNA Eraser (TaKaRa, Japan). Primer-BLAST was applied to design the specific divergent primers, which were used to amplify the circular transcripts by head-to-tail splicing. All primers were synthesized by Realgene (Nanjing, China). After the optimal annealing temperatures were determined, qPCR was performed on the Life Tech-ViiA7 system (Applied Biosystems, USA) using PowerUP SYBR Green Master Mix (Applied Biosystems, USA) to measure the relative expression levels of circRNAs. To reduce the experimental random error, samples were loaded in triplicate and each well was treated identically. Glyceraldehyde phosphate dehydrogenase (GAPDH) was used as internal control, and the relative expression levels of circRNAs were calculated with 2^-△△Ct^ method. Moreover, to guarantee the accuracy of the results, all data are represented as the means ± standard deviation (SD) of three independent experiments.

### Functional enrichment analyses

DAVID Bioinformatics Resources 6.8 was used to analyze the parental gene functions of the differentially expressed circRNAs. Gene Ontology (GO) analysis of the parental genes was conducted based on biological processes, cellular components and molecular functions (https://www.geneontology.org/). And Kyoto Encyclopedia of Genes and Genomes (KEGG, https://www.genome.jp/kegg) was used to analyze the related biological pathways. The -log (*P*-value) was used as the enrichment score indicating the significance of correlation.

### Annotation of circRNA/miRNA interactions

The miRanda (https://www.microrna.org/microrna/home.do/) was used to predict circRNA/miRNA interactions. Using the database, we searched for miRNA response elements (MREs) on circRNAs and selected miRNAs according to the seed matching sequences.

### Statistical analyses

Statistical analyses were conducted by SPSS 18.0 and GraphPad prism 5.0. All data are shown as mean ± SD. The student’s *t*-test was used to evaluate the differences between the experimental groups. Differences with *P* < 0.05 were considered statistically significant.

## Results

### CircRNA expression profiling in umbilical cord blood exosomes from PE patients compared with controls

**Fig. 1:**
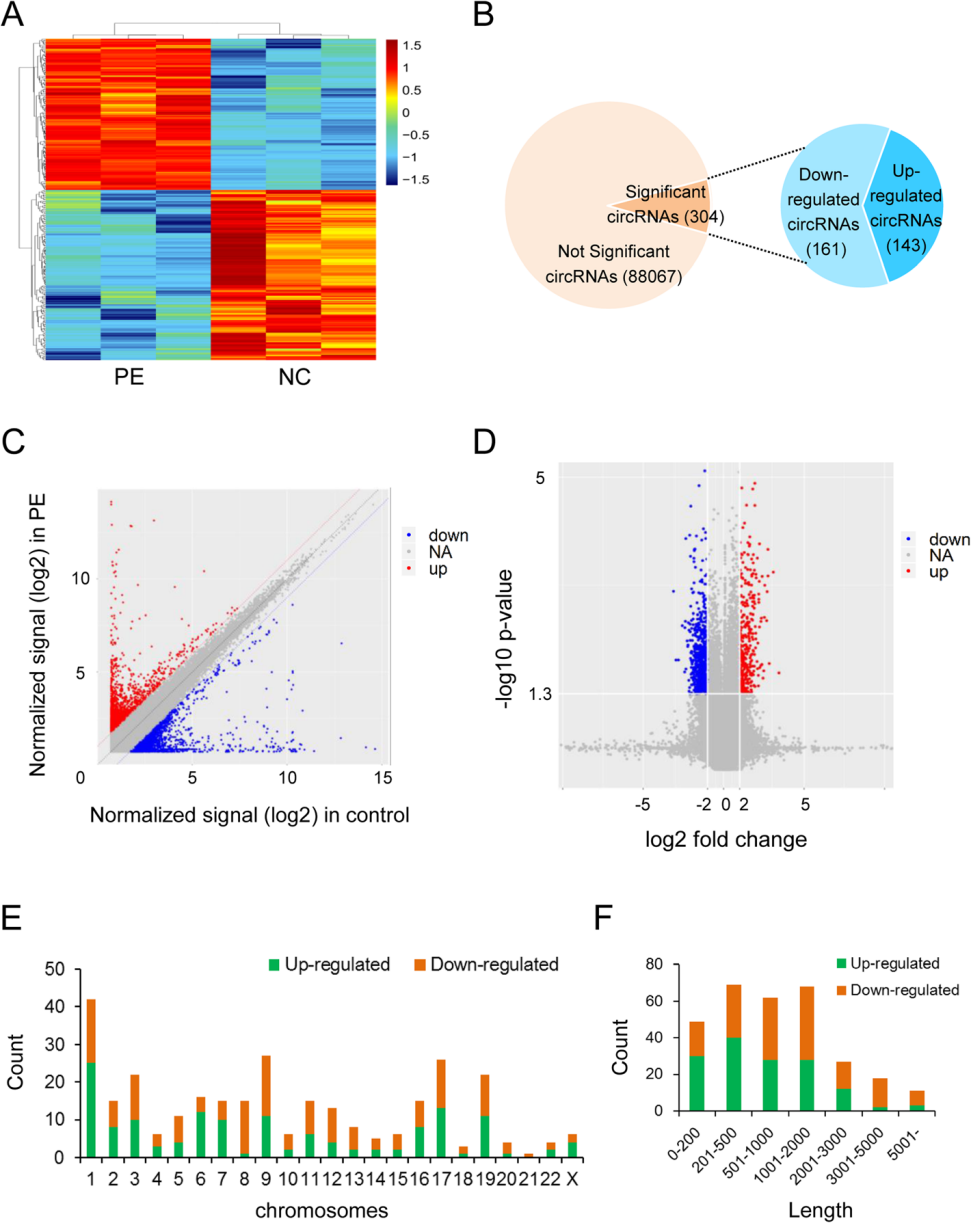
CircRNA expression profiling in umbilical cord blood exosomes from PE patients compared with controls. **a**: Clustered heat map analysis of differentially expressed circRNAs; **b**: The total circRNAs detected by microarray and differentially expressed circRNAs between two groups; **c**: Scatter plots of circRNAs signal values; **d**: Volcano plots visualizing the differentially expressed circRNAs; **e**: Chromosome distributions of the differential circRNAs; **f**: Length distributions of the differential circRNAs

We firstly performed a primary analysis of the microarray data to investigate the basic characteristics of all the circRNAs in umbilical cord blood exosomes. We assessed a total of 88,371 circRNAs, which are known from circBase (https://circrna.org/). Through hierarchical cluster analysis, we found that the levels of circRNA expression in umbilical cord blood exosomes were distinguishable between PE patients and the control group (Fig. 1a). Based on the screening criteria of fold change (FC) ≥ 2 and *P* < 0.05, a total of 304 circRNAs were screened as differentially expressed circRNAs. In the PE patients, 143 circRNAs were upregulated and 161 circRNAs were downregulated (Fig. 1b). Scatter plot and volcano plot were used to show circRNAs differentially expressed and circRNAs significantly differentially expressed, respectively (Fig. 1c and Fig. 1d). Table 1 lists the top 20 up and downregulated circRNAs. The results showed that the expression of circRNAs in cord blood exosomes was different between PE patients and the control group. Furthermore, Fig. 1e describes the chromosomal distribution of differentially expressed circRNAs and Fig. 1f shows that the lengths of the differential circRNAs were mostly shorter than 2000 bp.

**Table 1:** The top 20 up-regulated and down-regulated circRNAs between PE patients and controls

circRNA _ID	Chr	Length	Host gene	Normalized signal (log2)						Fold change	***P***	Regulation
				PE- sample1	PE- sample2	PE- sample3	NC- sample1	NC- sample2	NC- sample3			
circ_0077259	6	336	CGA	5.14	5.06	5.15	0.90	2.56	0.88	10.83	2.20E-02	up
circ_0077260	6	521	CGA	3.93	3.91	4.54	1.36	0.91	0.89	8.50	4.22E-04	up
circ_0081930	7	164	LAMB1	4.64	4.74	4.37	2.36	0.89	1.65	7.14	1.52E-02	up
circ_0043597	17	669	KRT19	3.88	4.24	4.08	1.64	1.17	1.09	6.76	4.90E-04	up
circ_0037771	16	166	NAGPA	3.63	3.36	3.87	0.73	0.77	1.11	6.74	1.72E-04	up
circ_0075840	6	995	GPLD1	4.20	4.27	4.31	1.97	2.27	1.20	5.21	1.58E-02	up
circ_0042232	17	126	MPRIP	3.75	4.60	3.99	2.20	1.40	1.75	5.06	2.55E-03	up
circ_0090100	X	525	SAT1	4.38	4.12	4.44	1.64	2.37	1.90	5.00	2.90E-03	up
circ_0013777	1	2640	NOTCH2	3.70	3.51	3.68	1.90	0.88	0.91	4.98	1.64E-02	up
circ_0081910	7	2197	LAMB1	4.51	4.59	4.89	2.45	2.60	2.27	4.68	1.38E-04	up
circ_0036082	15	4057	PAQR5	1.56	0.93	0.74	3.84	4.06	4.67	8.66	8.98E-04	down
circ_0042333	17	416	TOP3A	0.70	0.71	0.72	3.24	3.27	4.26	7.79	1.33E-02	down
circ_0062444	22	4291	PPM1F	0.75	0.76	0.75	3.66	2.88	3.94	6.99	1.32E-02	down
circ_0079385	7	245	ZDHHC4	2.13	1.06	1.52	4.02	3.79	4.66	5.93	3.42E-03	down
circ_0076206	6	1290	MTCH1	0.71	1.26	1.43	3.16	3.27	4.02	5.19	2.88E-03	down
circ_0066266	3	314	FAM116A	0.97	0.94	0.93	3.26	3.25	3.38	5.10	1.12E-04	down
circ_0004552	19	338	CARM1	2.55	0.90	1.42	3.61	3.94	4.66	5.06	1.89E-02	down
circ_0011389	1	1136	EIF3I	0.87	0.74	1.78	3.78	3.24	3.22	4.69	7.77E-03	down
circ_0008808	15	772	ARIH1	0.92	0.92	2.46	3.64	3.56	3.99	4.33	3.92E-02	down
circ_0029899	13	414	USPL1	1.06	1.29	0.98	3.39	3.06	3.20	4.31	9.83E-05	down
circ_0077259	6	336	CGA	5.14	5.06	5.15	0.90	2.56	0.88	10.83	2.20E-02	up
circ_0077260	6	521	CGA	3.93	3.91	4.54	1.36	0.91	0.89	8.50	4.22E-04	up
circ_0081930	7	164	LAMB1	4.64	4.74	4.37	2.36	0.89	1.65	7.14	1.52E-02	up
circ_0043597	17	669	KRT19	3.88	4.24	4.08	1.64	1.17	1.09	6.76	4.90E-04	up
circ_0037771	16	166	NAGPA	3.63	3.36	3.87	0.73	0.77	1.11	6.74	1.72E-04	up
circ_0075840	6	995	GPLD1	4.20	4.27	4.31	1.97	2.27	1.20	5.21	1.58E-02	up
circ_0042232	17	126	MPRIP	3.75	4.60	3.99	2.20	1.40	1.75	5.06	2.55E-03	up
circ_0090100	X	525	SAT1	4.38	4.12	4.44	1.64	2.37	1.90	5.00	2.90E-03	up
circ_0013777	1	2640	NOTCH2	3.70	3.51	3.68	1.90	0.88	0.91	4.98	1.64E-02	up
circ_0081910	7	2197	LAMB1	4.51	4.59	4.89	2.45	2.60	2.27	4.68	1.38E-04	up
circ_0036082	15	4057	PAQR5	1.56	0.93	0.74	3.84	4.06	4.67	8.66	8.98E-04	down
circ_0042333	17	416	TOP3A	0.70	0.71	0.72	3.24	3.27	4.26	7.79	1.33E-02	down
circ_0062444	22	4291	PPM1F	0.75	0.76	0.75	3.66	2.88	3.94	6.99	1.32E-02	down
circ_0079385	7	245	ZDHHC4	2.13	1.06	1.52	4.02	3.79	4.66	5.93	3.42E-03	down
circ_0076206	6	1290	MTCH1	0.71	1.26	1.43	3.16	3.27	4.02	5.19	2.88E-03	down
circ_0066266	3	314	FAM116A	0.97	0.94	0.93	3.26	3.25	3.38	5.10	1.12E-04	down
circ_0004552	19	338	CARM1	2.55	0.90	1.42	3.61	3.94	4.66	5.06	1.89E-02	down
circ_0011389	1	1136	EIF3I	0.87	0.74	1.78	3.78	3.24	3.22	4.69	7.77E-03	down
circ_0008808	15	772	ARIH1	0.92	0.92	2.46	3.64	3.56	3.99	4.33	3.92E-02	down
circ_0029899	13	414	USPL1	1.06	1.29	0.98	3.39	3.06	3.20	4.31	9.83E-05	down

### Validation of differentially expressed circRNA by qPCR

**Fig. 2:**
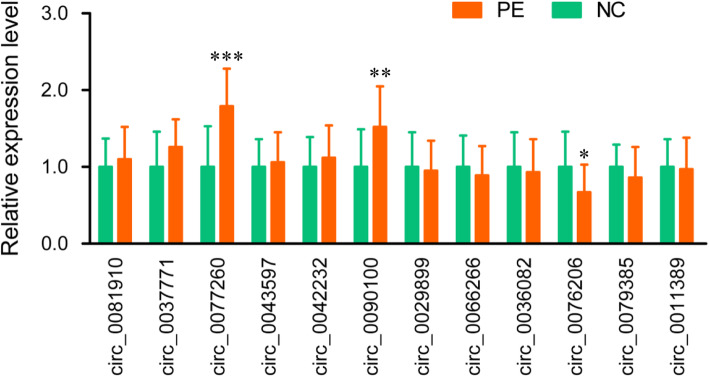
Validation of differentially expressed circRNA by qPCR. * *P* < 0.05, **P < 0.01 and *** *P* < 0.001

Based on relatively high abundance, FC ≥ 4, *P* < 0.01, and their host genes, we selected 12 candidate circRNAs to validate their expression in umbilical cord blood exosomes from additional 20 PE patients and 20 controls, including 6 up-regulated circRNAs (circ_0081910, circ_0037771, circ_0077260, circ_0043597, circ_0042232 and circ_0090100) and 6 down-regulated circRNAs (circ_0029899, circ_0066266, circ_0036082, circ_0076206, circ_0079385 and circ_0011389). In parallel with the microarray data, qPCR results showed that the expression of circ_0077260 and circ_0090100 were increased, and the expression of circ_0076206 were decreased in PE patients (Fig. 2).

### GO and KEGG pathway analysis of the circRNA parental genes

**Fig. 3:**
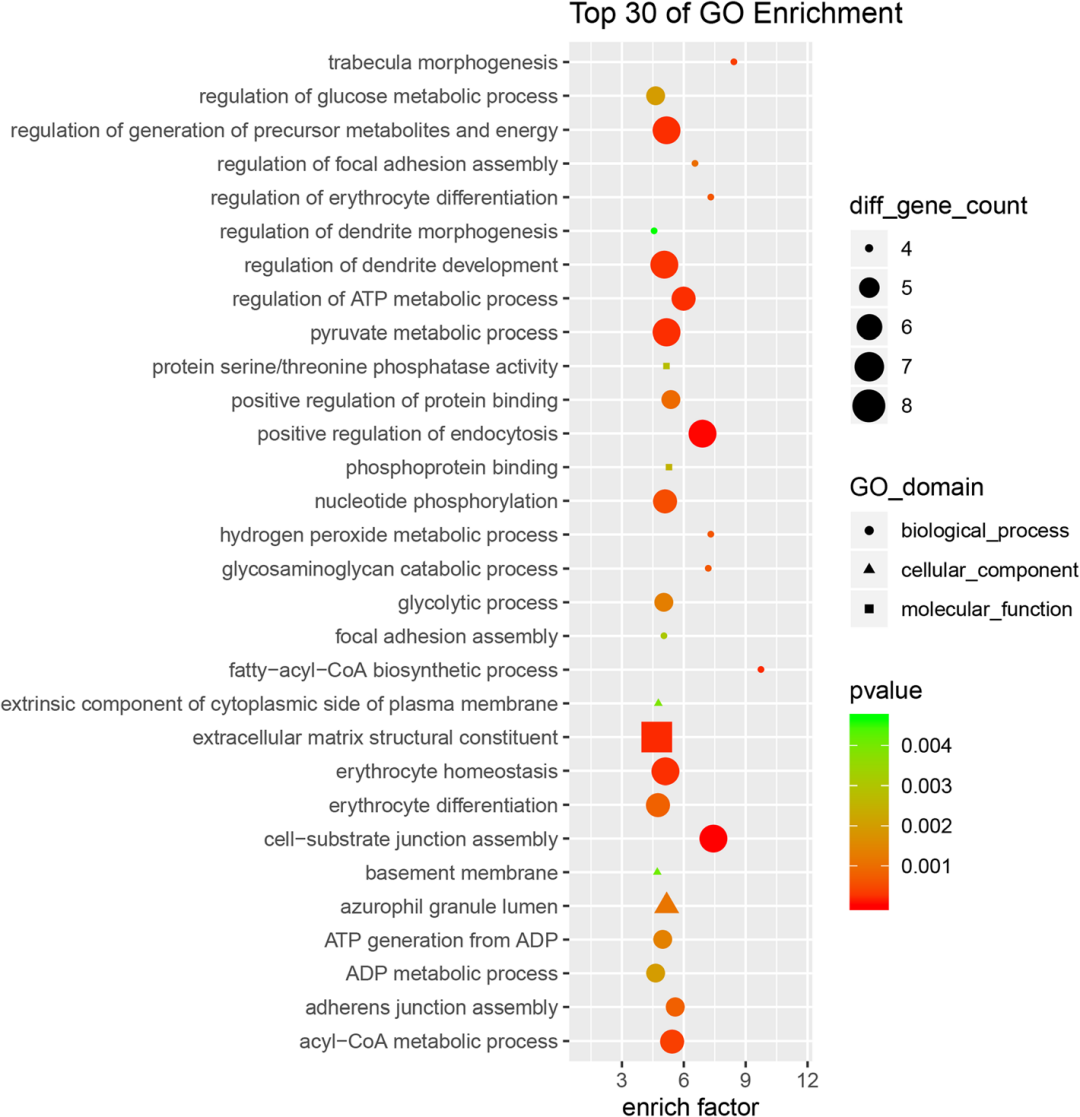
GO analysis of the differential circRNA parental genes

The biological functions of these differentially expressed circRNAs were detected by GO and KEGG pathway analysis. In GO analysis, the number of parental genes corresponding to GO entries was determined, and the -log (*P*-value) was taken as enrichment score. For biological process, the terms that contained the most genes were cellular process (GO:0009987, count = 180) and single-organism process (GO:0044699, count = 163), and the most significantly enriched term was cell-substrate junction assembly (GO:0007044, *P* = 1.73E-05). For cellular component, the terms that contained the most genes were cell (GO:0005623, count = 189) and cell part (GO:0044464, count = 182), and the most significantly enriched term was focal adhesion (GO:0005925, *P* = 2.30E-05). And for molecular function, the terms that contained the most genes were binding (GO:0005488, count = 179) and catalytic activity (GO:0003824, count = 114), and the most significantly enriched term was catalytic activity (GO:0003824, *P* = 8.90E-06) (Fig. 3). All mRNAs annotated involved in these GO terms were listed in Table S2.

**Fig. 4:**
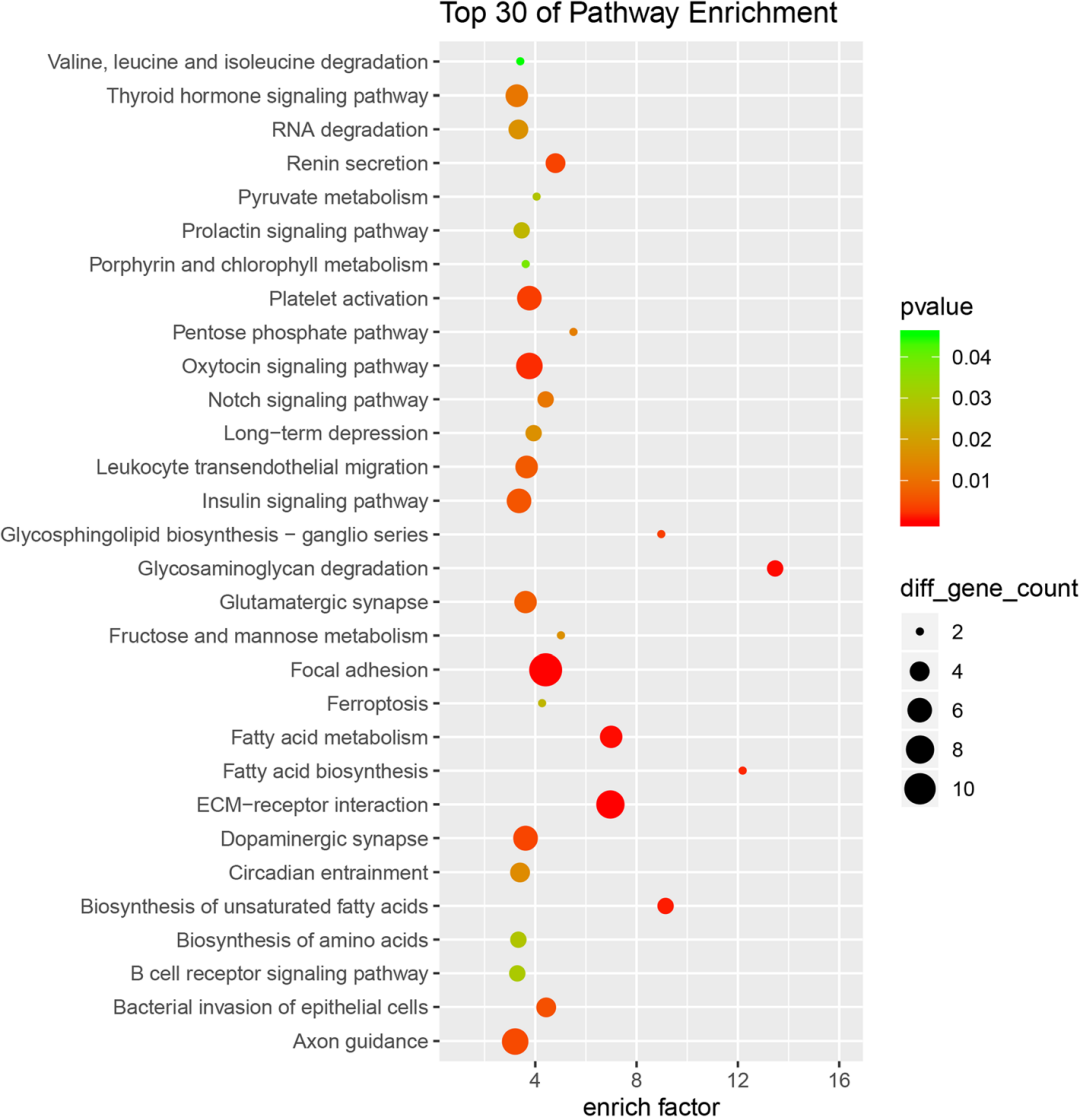
KEGG pathway analysis of the differential circRNA parental genes

**Fig. 5:**
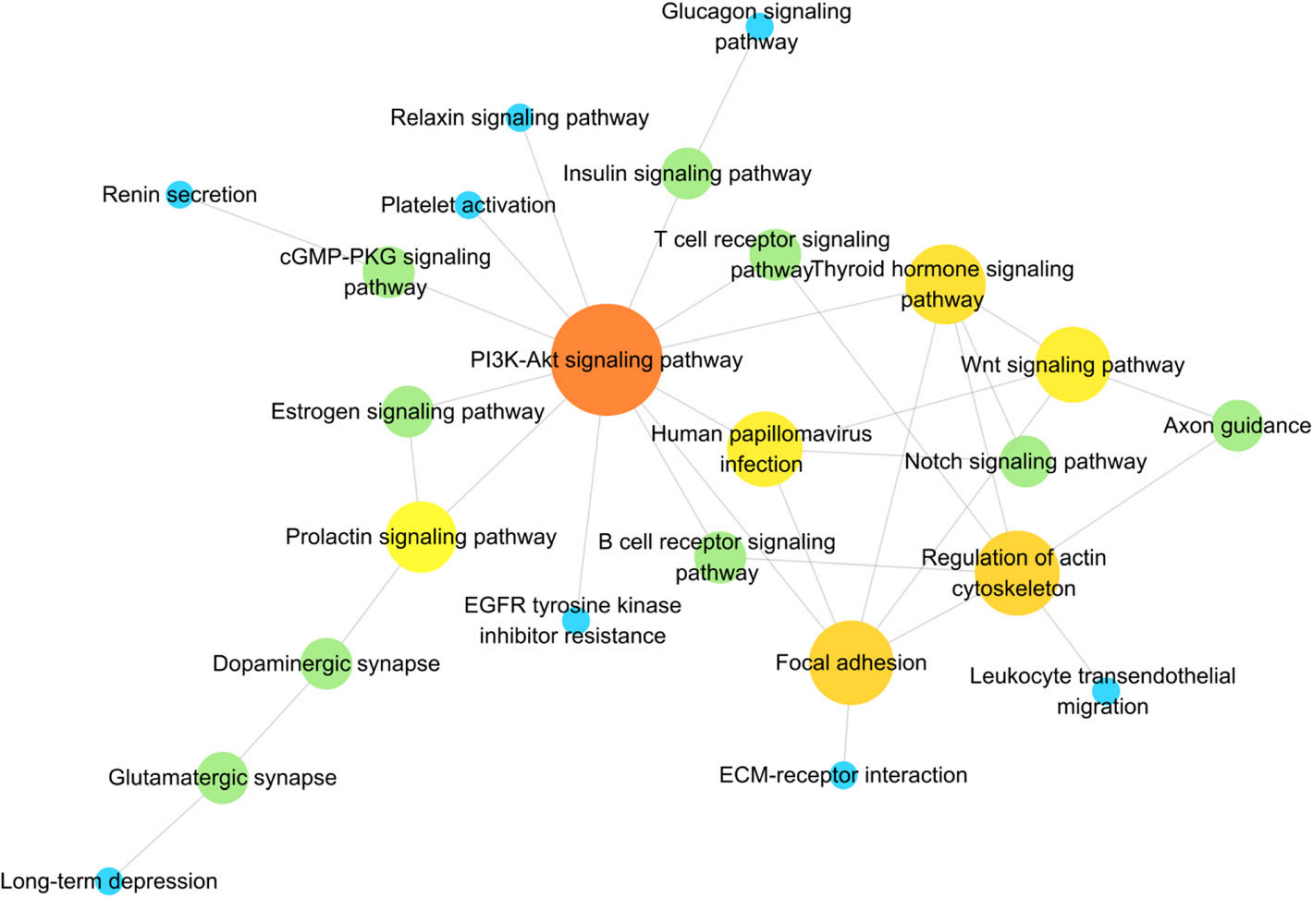
Interaction and overlaps of the differential circRNA parental genes among significantly enriched pathways

Furthermore, the KEGG results indicated that differentially expressed circRNAs were involved in ECM-receptor interaction (hsa04512), focal adhesion (hsa04510), glycosaminoglycan degradation (hsa00531), fatty acid metabolism (hsa01212), fatty acid biosynthesis (hsa00061) and Notch signaling pathway (hsa04330) (Fig. 4). All mRNAs annotated involved in these pathways were listed in Table S3. Then, we used these pathways to construct a pathway network to investigate the key pathways in PE. As is shown in Fig. 5, the exchanges with these pathways largely depended on the existence of PI3K-Akt signaling pathway.

### Prediction of circRNA/microRNA interactions

Studies have shown that RNAs regulate each other with miRNA response elements (MREs) and this mechanism is called the hypothesis of “competing endogenous RNA (ceRNA)” [21]. CircRNAs could act as ceRNA molecules or effective miRNA sponges to regulate the expression, transcription, and protein synthesis of miRNA-targeted genes [21]. The miRanda was used to predict the interactions between the differentially expressed circRNAs and miRNAs based on MREs. Based on the criteria of max score ≥ 140 and max energy ≤ − 20, 2226 miRNAs were found to pair with 302 differentially expressed circRNAs (Table S4); the lower the maximum energy, the more significant the correlation. The results suggest that circRNAs may be involved in the pathogenesis of PE through interactions with PE incidence-related miRNAs.

## Discussion

Exosomal circRNAs have become the focus of research in recent years because of their distant regulatory potency [15]. In this study, the differential expression profile of circRNA in umbilical cord blood exosomes of PE patients was constructed for the first time, providing a basis for subsequent studies on the relationship between exosomal circRNA and PE development. Based on the microarray data, we identified 304 differentially expressed circRNAs in umbilical cord blood exosomes of PE patients when compared with normal controls, including 143 upregulated circRNAs and 161 downregulated circRNAs, which indicated that the expression pattern of exosomal circRNAs in PE patients was different from that in controls.

In subsequent validation experiments, the expression of exosomal circ_0077260 and circ_0090100 were significantly increased, and the expression of exosomal circ_0076206 were significantly decreased in PE samples. Growing evidence has shown that circRNAs can regulate parental gene expression through diverse mechanisms, such as transcription and splicing regulation, miRNA sponges, mRNA traps, translational modulation, and post-translational modification [22]. The parental genes of circ_0077260, circ_0090100 and circ_0076206 are CGA, SAT1 and MTCH1, respectively. Of these parental genes, CGA encodes for the common alpha subunit of four glycoprotein hormones, hCG (human chorionic gonadotropin), LH (luteinizing hormone), FSH (follicle-stimulating hormone) and TSH (thyroid-stimulating hormone) [23]. Previous studies have found that α-hCG is correlated with PE [24], and CGA was differentially expressed in placenta tissue among late-onset PE, early-onset PE and healthy controls [25, 26]. CGA is also considered as a novel estrogen receptor response gene in breast cancer and an outstanding candidate marker for predicting response to endocrine therapy [27]. Further studies are needed to determine whether the association among circ_0077260, CGA and estrogen is involved in the pathogenesis of PE.

GO and KEGG pathway analyses were performed to predict the biological functions and potential pathways of these differential circRNAs. Notablely, several pathways were found to be significantly enriched, such as focal adhesion, glycosaminoglycan degradation, fatty acid metabolism, fatty acid biosynthesis and Notch signaling pathway. It is well known that focal adhesion is crucial to trigger cell adhesion and many other cellular processes including cell migration, spreading and proliferation [28], which are important in PE development. And localization studies in placental tissues have showed that cytotrophoblasts in all stages of differentiation express focal adhesion kinase [29]. In terms of metabolic process, PE has been demonstrated to be associated with increased insulin resistance, hypertriglyceridemia, high circulating free fatty acids, low high-density lipoprotein particles, and high maternal and fetal plasma amino acid concentrations [30]. These metabolic alterations may contribute to the pathophysiology of the syndrome and may also influence fetal growth. For Notch signaling pathway, defects in this pathway would have adverse effect on placentation. And it has been suggested that Notch pathway down-regulation is associated with PE [31]. Further constructed pathway network showed that the exchanges with these pathways largely depended on the existence of PI3K-Akt signaling pathway. The PI3K-Akt signaling pathway has been demonstrated to be a critical pathway mediating the growth-factor-dependent regulation of trophoblast growth and invasion [32]. The insufficient invasion of trophoblasts is known to be correlated with the development of PE [32]. Together, the altered circRNAs are associated with metabolic process, trophoblast growth and invasion related signaling pathways. Efficient biomarkers underlying these pathways need to be further investigated.

A large amount of evidence have indicated that exosomal circRNAs could act as ceRNA molecules or efficient miRNA sponges to regulate miRNA-targeted gene expression, transcription and protein synthesis [33–35]. The circRNAs may have many miRNA binding sites that competitively bind to miRNAs, and then alleviate the inhibitory effects of miRNAs on target molecules [21]. In this study, through circRNA/miRNA interactions analysis, we found that most of the exosomal circRNAs had miRNA binding sites, and some miRNAs were associated with PE. For example, miR-17-3p, miR-197, miR-424, miR-431 and miR-483 were reported to be aberrantly expressed in preeclamptic placenta [36–40]. miR-17-3p and miR-424-5p were matched with circ_0077260, which was verified to upregulated in the umbilical cord blood exosomes of PE patients; miR-197-5p and miR-431-5p potentially binds to downregulated circ_0076206; whereas miR-424-5p and miR-483-3p potentially matched with upregulated circ_0090100. Specifically, exosomal miR-486-1-5p and miR-486-2-5p were reported to be upregulated in PE pregnancy compared with normal pregnancy [41]. And miR-486-5p was matched with downregulated circ_0076206. In addtion, miR-885-5p was increased in plasma from PE patients compared with healthy pregnant women, and it was released into circulation mainly inside exosomes [42], whereas miR-885-5p potentially matched with upregulated circ_0077260. Therefore, we hypothesized that the role of exosomal circRNAs in PE development may be related to miRNA-mediated effects. The potential mechanism of the circRNA-miRNA-target gene interaction in PE is worthy of further study.

## Conclusions and prospect

Our study firstly showed that exosomal circRNAs are aberrantly expressed in the umbilical cord blood of PE patients. The potential roles of these differentially expressed circRNAs and their interactions with miRNAs were further predicted through bioinformatics analysis, highlighting the importance of exosomal circRNAs in the pathogenesis of PE and providing a basis for further studies on function and mechanism of exosomal circRNAs in PE development. Studying the structure of circRNAs may lead to the development of effective artificial sponges to regulate the progression of disease. Artificial miRNA sponge technology, as an effective and stable miRNA inhibitor, may become a new direction of RNA gene therapy. It inhibits the expression of other miRNAs at the same time and produces a more persistent inhibition. In addition, because the expression profile of exosomal circRNAs in PE patients is different from that in the healthy group, combinations of exosomes and circRNAs are beneficial for their clinical application as diagnostic and prognostic biomarkers. Larger cohort studies are warranted to demonstrate that exosomal circRNAs are clinically applicable biomarkers.

## Supplementary Information

**Additional file 1: Table S1** Clinical data for the GDM patients and normal controls. **Table S2** GO enrichment analysis of the circRNA parental genes. **Table S3** KEGG enrichment analysis of the circRNA parental genes. **Table S4** Prediction of circRNA/microRNA interactions

## Data Availability

The raw data of microarray in this study has been uploaded to GEO repository (GEO accession number is GSE166846, https://www.ncbi.nlm.nih.gov/geo/query/acc.cgi?acc=GSE166846). And all datasets generated for this study are included in the manuscript and the supplementary files.

## Abbreviations

CircRNAs: Circular RNAs
PE: Preeclampsia
GO: Gene Ontology
KEGG: Kyoto Encyclopedia of Genes and Genomes
qPCR: Quantitative real-time PCR
GAPDH: Glyceraldehyde phosphate dehydrogenase
MREs: miRNA response elements
SD: Standard deviation
FC: Fold change
ceRNA: Competing endogenous RNA
hCG: Human chorionic gonadotropin
LH: Luteinizing hormone
FSH: Follicle-stimulating hormone
TSH: Thyroid-stimulating hormone
